# Instruments Measuring Self-Care in Children and Young Adults With Chronic Conditions: A Systematic Review

**DOI:** 10.3389/fped.2022.832453

**Published:** 2022-03-28

**Authors:** Valentina Biagioli, Giuseppina Spitaletta, Valeria Kania, Rachele Mascolo, Orsola Gawronski, Annachiara Liburdi, Giulia Manzi, Michele Salata, Ercole Vellone, Emanuela Tiozzo, Immacolata Dall’Oglio

**Affiliations:** ^1^Professional Development, Continuing Education and Research Service, Bambino Gesù Children’s Hospital, IRCCS, Rome, Italy; ^2^Department of Paediatric Emergency, Bambino Gesù Children’s Hospital, IRCCS, Rome, Italy; ^3^Pediatric Semi-Intensive Care Area/Unit, Bambino Gesù Children’s Hospital, IRCCS, Rome, Italy; ^4^Rheumatology Unit, Bambino Gesù Children’s Hospital, IRCCS, Rome, Italy; ^5^Department of Biomedicine and Prevention, University of Rome “Tor Vergata,” Rome, Italy

**Keywords:** self-care, self-management, instruments, chronic disease, pediatric, adolescent, young adult, parent

## Abstract

Children and young adults (CYAs) with chronic conditions need to engage in self-care to improve their quality of life. This study aimed to retrieve the literature on instruments to assess self-care in CYAs living with chronic conditions and evaluate the psychometric proprieties of the instruments retrieved. A systematic literature review was conducted on six databases to identify peer-reviewed papers that described or used an evaluation instrument of self-care in CYAs with chronic conditions. Twenty-three articles describing 11 instruments of self-care were identified. Five instruments (45.45%) were developed for specific diseases, while six (54.54%) for various chronic illnesses. Most of the instruments were focused on treatment adherence within self-care maintenance (i.e., behaviors to maintain illness stability), excluding the monitoring of clinical parameters or the management of exacerbations. This review provides an overview of available instruments that measure self-care in CYAs with chronic conditions, which health professionals could use for patient education.

## Introduction

Chronic diseases are defined broadly as conditions that last for a year or longer and require ongoing medical attention or limit activities of daily living or both (1). The prevalence of pediatric-onset chronic diseases is gradually increasing around the world (2), contributing to both morbidity and mortality (3). The number of children and young adults (CYAs), children aged 0–24 years (4, 5), living with a chronic condition is growing due to higher survival rates (6). In the United States, 25% of the pediatric population is affected by a chronic condition and 5% by multiple chronic conditions (7). In Europe, in 2016, 16% of the population aged between 16 and 29 had a long-standing health problem (8). In Italy, 91.8% of children aged 0–14 are in good health, 9.6% have one or more chronic conditions, 1.6% suffer from two or more chronic illnesses (9). In addition, children with complex conditions have to deal with multiple transitions across providers and care settings (10), and those requiring technology support and home care bear even higher costs (11–13). Furthermore, long-term chronic conditions have a strong impact on wellbeing and require ongoing management over a period of years or decades (14).

In the pediatric population, the most common pediatric chronic conditions, including those with medical complexity (15), are asthma, cystic fibrosis, type 1 diabetes mellitus, and chronic lung disease (16). In particular, children with the highest levels of medical complexity are estimated to be about 0.4–0.7% of all US children (17). Therefore, it is important to promote the quality of life of CYAs with chronic conditions and their family members. This requires a life-long process of self-care or self-management to preserve and improve personal wellbeing, to maintain a good health-related quality of life, and to reduce health costs (18, 19).

The concepts of self-care and self-management have been used with considerable overlap and interchangeably among scholars (20). Self-management refers to the process that individuals with a health problem intentionally use to gain control of their disease, in partnership with health professionals (21). Self-care is a more encompassing concept, referring to patients’ ability and performance of activities to achieve, maintain, and promote optimal health and wellbeing, including monitoring and managing acute and chronic health conditions (22, 23). WHO defines self-care as “the ability of individuals, families and communities to promote health, prevent disease, maintain health, and to cope with illness and disability with or without the support of a healthcare provider” (24). According to Riegel and Dickson (25), self-care is a naturalist decision-making process based on patient experience (25).

In particular, the Middle-Range Theory of Self-Care of Chronic Illness identifies behaviors of self-care maintenance, characterized by those actions performed to maintain chronic condition stable (e.g., taking medications as prescribed); self-care monitoring, concerning all those behaviors performed to keep signs and symptoms under control (e.g., monitoring weight); and self-care management, concerning the reaction to symptoms when they occur (e.g., call the healthcare provider in case of fever) (26, 27). However, this Middle-Range Theory was developed for adults. In the pediatrics, especially for CYAs, few theoretical models have been described, such as the new comprehensive model of self-care in CYAs (28). This model emphasized that self-care is a very broad concept since it not only includes personal skills but also healthcare actions provided by others. Others include informal caregivers (parents, relatives, friends, volunteers) who play a crucial role in chronic patient care, but also formal caregivers (healthcare professionals) who provide specific professional support to families in terms of care management (28). Healthcare professionals cooperate with the patient and/or the family who maintain, if possible, the responsibility for their own care (29).

Self-care and quality of life, distress, and depression are interrelated (18). On the one hand, better self-care is associated with positive outcomes, such as more adequate disease control, greater patient safety, higher quality of life, and better personal development, which may lead to lower depression and distress (28, 30). On the other hand, psychological aspects can also be considered as influencing factors; for example, if CYAs are depressed, then they are more likely to neglect self-care behaviors (31, 32). Moreover, healthcare systems admit that self-care has a positive impact on reducing chronic diseases and on reducing health costs (33). Indeed, in general, chronic diseases requires a great amount of human and economic resources (34). Managing chronic diseases requires specialized professional competences and health facilities suited to the health care pathways. Therefore, the chronic diseases during childhood have a strong social impact (35).

In addition, the health consequences are related to the child’s age at the onset of chronic alteration (36). Children with chronic diseases occurring during childhood showed a different outcome compared to those in which the diseases onset during their adolescence. Indeed, many aspects of adolescent daily life require a life-long process of self-care such as the need for precisely scheduled daily medications, consumption of special dietary products, regular physical exercise, regular visits to healthcare providers and monitoring of blood glucose levels (37).

Furthermore, adolescents with a chronic disease may deal with the burden of independence incapability and the need to ask for support from parents and other caregivers for most of their daily activities (37). Parents should encourage adolescents to develop self-esteem and avoid an excessively protective attitude (38). Adolescence is a key development period for establishing lifelong health-related behaviors (39). Furthermore, patients with complex chronic diseases, along with developmental changes in adolescence, face challenges related to their health-related quality of life (40).

There is evidence that self-care actions have a positive impact on the health of CYAs with complex chronic diseases, such as diabetes and fibrosis cystic (28, 41). Therefore, it is essential that these patients perform self-care (38, 42). The higher educational level of the population has generated a higher demand for specific information and education regarding healthcare topics (43). This demand has caused an increase of CYAs’ care competency for their own health and wellbeing (43). Assessing self-care in the pediatric population with chronic diseases may contribute to improve self-care activities and address any deficiencies.

Therefore, the aims of this study were: (a) to retrieve and describe the literature on instruments (scales or questionnaires) that assess self-care in CYAs living with chronic conditions; and (b) to evaluate the psychometric proprieties of the retrieved instruments that assess self-care in CYAs with chronic conditions.

## Methods

### Search Strategy

A systematic review was conducted to explore studies that described self-care scales for pediatric patients with chronic diseases. Search procedures followed the Preferred Reporting Items for Systematic Reviews and Meta-Analyses (PRISMA) guidelines for writing systematic reviews (44). The review was conducted through six databases: PubMed, Scopus, CINAHL, Embase, PsycInfo, and the Cochrane Library. In addition, a manual search was carried out to broaden the search. The study selection was conducted in July 2021. The main keywords were “self-care,” “self-management,” “Scale,” “Questionnaire,” “Chronic Disease,” “Pediatric,” “Adolescent,” “Young Adult,” “Parents.” Boolean operators—NOT, AND, OR—were also used to narrow and widen the search. The search was conducted by two reviewers independently. The search strategy is described in Supplementary Material.

### Eligibility Criteria

The review included all types of peer-reviewed papers with no limits of time or language. Eligible studies for inclusion had to meet the following criteria: (a) any study published on a peer-reviewed journal; (b) patients with chronic diseases or complex chronic diseases; (c) patients aged between 0 and 24 years; (d) studies that described or used a self-care evaluation scale; (e) studies describing self-care in children or young adults and/or the parental role; (f) studies in any language describing self-care evaluation instruments.

The exclusion criteria were: (a) papers that did not include instruments that evaluated self-care; (b) self-care scales not developed for the population included in this review; (c) unavailable full-texts; (d) scales that did not describe self-care activities; (e) papers published in journal that were not peer-reviewed; (f) scales did not include at least one of the self-care dimensions (self-care maintenance, self-care monitoring, self-care management); (g) studies that evaluated only self-efficacy.

### Study Selection

Firstly, duplicate records were identified and removed. Secondly, titles and abstracts were screened by two independent authors. The full texts of potential eligible studies were read to determine if the papers were eligible. In case of disagreement between the two authors, a third author was involved to make the final decision.

### Data Extraction and Synthesis

The following data were extracted: authors and year of publication; country where the study was conducted; aim; study design; population (patient and/or parents age); type of chronic diseases and if mental diseases were included; scales or questionnaires; administration method; timing of administration; whether the instrument was validated; and findings. To describe and synthesize information on every instrument included in this review, the included papers were examined by focusing on the following information: name of the scale; description of the scale or part of it; original author and year; authors who included the scale in their paper; whether the scale was original or adapted; language; whether the entire scale or only one of its dimensions were used; patients’ age; chronic diseases; self-care maintenance, self-care monitoring, self-care management; the population that responded to questionnaire (patients, parents, or both), the way the scale and/or questionnaire was administered, and the conceptual model (25). The psychometric characteristics of each included instrument were analyzed using the COSMIN criteria (45). In addition, two researchers independently investigated the dimension of self-care reported in each scale (self-care maintenance, self-care monitoring, and self-care management) according to the Middle-Range Theory of Self-Care of Chronic Illness (26).

## Results

**FIGURE 1:**
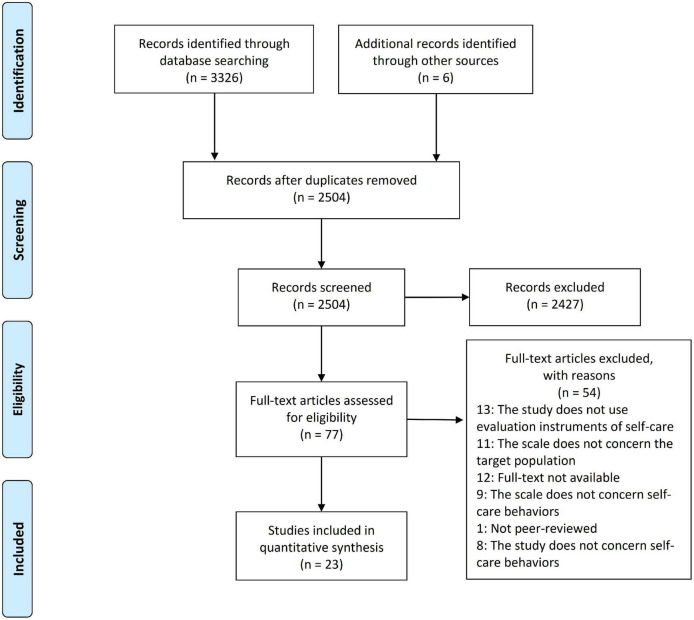
The Preferred Reporting Items for Systematic Review and Meta-Analysis (PRISMA) flow diagram showing the study selection process.

The study selection process is shown in Figure 1. The initial search identified 3,326 records across the six databases and six articles after a manual search. After removing the duplicates, 2,545 articles were reviewed by reading the title and abstract and 2,468 were excluded because they did not meet the inclusion criteria. The full texts of the remaining 77 articles were read and, of these, 23 papers were included in the final review and analysis. The reasons for the exclusion of 54 papers are reported in Figure 1.

### Characteristics of the Included Studies

Most of the 23 studies included in this review were published in the last decade (*n* = 20; 86.95%) and mainly conducted in North America (*n* = 15; 65.21%), followed by Asia (*n* = 5; 21.73%), Europe (*n* = 2; 8.69%), and Mexico (*n* = 1; 4.34%) (Table 1). Seven studies (30.43%) used a cross-sectional approach, two (8.69%) were pilots, one (4.34%) was a longitudinal observational study, one (4.34%) used a qualitative design, and 12 (52.17%) did not report their study design. The age of the study samples ranged from two to 28 years, including children, adolescents and/or young adults. The samples of six of the 23 included studies (26.08%) included both children and their parents, whereas one study (4.34%) only the parents.

**TABLE 1:** Characteristics of the articles included in the review.

Author-Year and Country	Aim of the study	Study design	Population (patient\parents, age) and chronic condition	Neuro developmental and/or mental disorder*	Measurement tool	Administration method and data collection	Results
Klinnert et al. (52) United States	This paper presents an assessment tool, the Family Asthma Management System Scale (FAMSS), evaluating the effectiveness of the family asthma management system	NR	30 mothers of children with asthma	NR	Family Asthma Management System Scale	Pediatric psychologists rated the audiotapes of the interviews using the FAMSS Rating Scales	The FAMSS is a good measure of the family’s ability to manage a chronic illness effectively
Patton et al. (38) United States	To describe the development and psychometric properties of a survey tool designed to evaluate children’s level of independence in treating their cystic fibrosis (CF)	NR	76 patients with cystic fibrosis (age = 4–17 years)	NR	Self-care Independence Scale	Self-administered, via mail and phone	The SCIS has acceptable internal consistency and good test-retest reliability. The construct validity of the SCIS was supported by positive correlations between patient age, number of years since diagnosis, and SCIS total scores.
Schilling et al. (49) United States	To report the development and testing of a new self-report measure to assess self-management of type 1 diabetes in adolescence (SMOD-A)	Qualitative descriptive study	515 adolescents with type 1 diabetes (age = 13–21 years)	NR	Self-Management of Type 1 Diabetes in Adolescence (the SMOD-A)	Self- administered, participants approached in clinic waiting rooms	The SMOD-A was a reliable, stable, and valid measure of self-management of type 1 diabetes in adolescents. Content validity (CVI = 0.93), acceptable subscale reliability (α = 0.71–0.85)
Modi et al. (50) United States	To describe the development and validation of a Pediatric Epilepsy Medication Self-Management Questionnaire (PEMSQ) for caregivers of children 2–14 years	NR	119 children with epilepsy (age = 2–14 years) and their families	NR	Pediatric Epilepsy Medication Self-Management Questionnaire	Self-administered, participants approached during follow-up clinic appointments	The PEMSQ showed strong psychometric properties, including good internal consistency across scales and construct validity with objective and subjective measures of adherence.
Giardini et al. (62) Italy	To present the Adherence Schedule in Transplantation (ASiT, in its three versions: ASiT-A, Adults; ASiT-PA, Proxy Adult and ASiT-PC, Proxy Child)	NR	56 adult patients, transplant recipients (liver, heart, lung, and kidney) and parents of children	NR	Adherence Schedule in Transplantation-Proxy Child (ASiT-PC)	Self-administered, participants approached in different hospital units	Within a clinical context the schedule tool could be foster communication about adherence and enhance patients’ personal limits and resources.
Sawicki et al. (65) United States	To develop the TRAQ, a measure of readiness for transition from pediatric to adult healthcare	NR	192 youths with special health care needs (age = 16–26 years)	Autism Spectrum Disorder or mild mental retardation; attention-deficit hyperactivity disorder, learning disabilities, behavior disorders, bipolar disorder	Transition Readiness Assessment Questionnaire —TRAQ	Self-administered, online through 2 sites	The TRAQ is a reliable measure for assessing skills in self-management and self-advocacy. The TRAQ may also be useful for YSHCN*, their caregivers, and clinicians as a tool to identify areas for patient education and track progress throughout the transition process. Each domain had a high internal consistency.
Ferris et al. (56) United States	To describe the development of the University of North Carolina (UNC) TRxANSITION Scale that measures the health-care transition and self-management skills in youth with chronic health conditions	NR	185 adolescents and emerging adults with different chronic illnesses (age = 12–22 years)	NR	UNC TRxANSITION Scale (Version 3)	Semi-structured, clinically feasible interview tool administered by trained professionals	The UNC TR(x)ANSITION Scale is a reliable and valid tool that has the potential to measure health-care transition skill mastery and knowledge in a multidimensional fashion. Inter-rater reliability was strong (*r* = 0.71) and item-total correlation scores were moderate to high. Content and construct validity were satisfactory.
Guo et al. (47) China	To assess diabetes self-management, depressive symptoms, quality of life and metabolic control in a cohort of youth with type 1 diabetes in mainland China.	Cross-sectional study as part of a multi-site longitudinal descriptive study.	136 youths with type 1 diabetes (age = 8–19 years)	NR	Chinese Version of Self-Report Measure of Self-Management of Type 1 Diabetes for Adolescents (C-SMOD-A)	Self- administered, by telephone	Self-management (Diabetes Care Activity subscale of C-SMOD-A) varied by socio-demographic characteristics. Girls had higher care self-management levels than boys (*p* = 0015), as did youths in two parent families (*p* = 0028). Youths who dropped out of school reported lower diabetes self-management levels than those still in school (*p* = 0003). Youths on intensive insulin treatment regimens had significantly better self-management compared to those not on intensive insulin treatment (*p* < 0001). Youths who were recruited from the CSU diabetes center had significantly better self-management than youths recruited from other hospital sites (*p* = 0001).
Sawicki et al. (60) United States	To assess Health care transition readiness using the TRAQ to understand associations between self-care beliefs, HCT readiness skills, and reports of HCT preparation among adolescents/young adults.	NR	79 youths with cystic fibrosis, diabetes and myelodysplasia/spina bifida (age = 16–25 years) and 52 parents	NR	TRAQ	Self- administered, data pooled from 2 surveys	High confidence in self-care espoused by youths and parents was belied by low reported readiness to manage many of the discrete tasks necessary for autonomous care, and by exceedingly low levels of preparation and planning for transition.
Cantú-Quintanilla et al. (53) Mexico	To provide further validation of UNC TRxANSITION Scale™ Version 3 in a Spanish version among Mexican youths with Chronic Kidney Disease or End-Stage Kidney Disease.	NR	163 youths with chronic kidney disease or end-stage kidney disease (age = 10–22 years)	NR	Spanish version of UNC TRxANSITION Scale™ Version 3	Administered by a trained psychologist	The Spanish Version of the UNC TRxANSITION Scale™ version 3 is a brief, reliable and clinically applicable tool that is easy to administer and has demonstrated initial validity in Mexican youths with CKD or ESKD.
Cohen et al. (54) United States	This paper examines the validity of the STARx Questionnaire, and includes examination of concurrent, predictive, and discriminant types of validity.	NR	252 AYA with different chronic conditions	NR	STARx Questionnaire (Successful Transition to Adulthood with Therapeutics)	Self- administered, via online survey or via paper-and-pencil formats	The strong validity of the STARx Questionnaire, in tandem with its strong reliability, indicated adequate psychometric properties for this generic self-reporting measure. These strong psychometric properties should contribute to the STARx being a viable measure of health care transition for both research and clinical purposes.
Ferris et al. (29) United States	The Self-Management and Transition to Adulthood with Rx = Treatment (STARx) Questionnaire was developed to collect information on self-management and HCT skills	Pilot study	194 youths, AYAs, with different chronic conditions, and their parents	NR	STARx	Self- administered, via paper and pencil or on-line	The STARx Questionnaire is a reliable, self-report tool with adequate internal consistency, temporal stability, and a strong, multidimensional factor structure. It provides an additional assessment strategy to measure self-management and transition skills in AYAs with chronic conditions.
Moynihan et al. (58) Canada	To refine and psychometrically test the Am I ON TRAC for Adult Care questionnaire.	Descriptive cross-sectional study.	200 adolescents with different chronic conditions (age = 12–19 years)	NR	Am I ON TRAC	Self- administered	Factor analysis of the knowledge items identified a 14-item unidimensional scale. Knowledge and behavior sub-scale scores increased with age, with a stronger relationship between knowledge and age. Psychosocial maturity correlated with both sub-scale scores, but had a stronger association with behavior. Psychosocial maturity and age had a weak but significant correlation suggesting age is a loose proxy for maturity.
Karahroudy et al. (48) Iran	To determine the relationship between demographic characteristics and self-management in adolescents with type 1 diabetes.	Descriptive-analytical cross-sectional study	426 adolescents with type 1 diabetes (age = 13–18 years)	NR	Self-Management of type 1 Diabetes in Adolescence (SMOD-A)	Self- administered, in the hospital or at home	The results showed that the presence of another diabetic member in the family leads to higher levels of self-management in some dimensions, including problem-solving, communication, and goals, yet to lower levels in some others, including collaboration with parents and diabetes care activities
Nazareth et al. (59) United States	To improve reliability and generalizability of the STARx and report initial reliability data on the STARx-P Questionnaire	NR	341 parents (89.4% mothers) and 455 children with kidney disease, inflammatory bowel disease, diabetes, cerebral palsy, sickle cell, and cystic fibrosis (mean age = 12.28 ± 2.53)	NR	STARx (Successful Transition to Adulthood with Therapeutics) STARx P	Self- administered, via e-mail	The current study shows the same factor structure for a parental version (STARx-P) with good internal reliability. Age was significantly correlated with all factors as well as the total score for the AYA patient reported STARx scores, while the parent report STARx-P was only significantly related to age for Factor 2: Self-management
Sawin et al. (46) United States	The purpose of this psychometric study was to evaluate the reliability and validity of the 17-item generic Adolescents Young Adult Self-Management and Independence Scale II (AMIS II).	NR	201 adolescents - young adults with spina bifida (age = 12–25 years) and 111 parents	NR	AMIS II	Structured interview rated by health care providers, via in-person or telephone interview	Exploratory factor analysis of parent data supported the Condition Self-Management and Independent Living Self-Management. CFA of AYA data confirmed these two factors and an overall scale with good fit statistics (GFI and CFI = 0.86 to −0.95; RMSEA = 0.057). Internal reliabilities ranged from o: = 0.72–0.89. Intraclass correlation analysis supported the stability of the instrument (ICC* parent report = 0.82, AYA report = 0.84). Concurrent validity was supported with low to moderate correlations IO six related but distinct variables.
Sheng et al. (2) China	To explore the relationships between FM, self-management and transition readiness, and quality of life (QoL), and identify the potential CYP or family factors influencing the relationships.	Cross-sectional design	268 patients, CYP, with diabetes, rheumatic disease, or renal disorder (age = 8–18 years) and their caregivers	NR	STARx	Self- administered, paper and pencil	This study was unique in that it identified FM*-related facilitators and barriers to CYP’s self-management and transition readiness skills and explored optimal mechanisms for the provision of family-focused transition support in health-care settings.
Zhong et al. (61) United States	To evaluate the roles of key individual, family, and illness characteristics on the levels of and gains in longitudinal healthcare transition readiness	Longitudinal observational study	566 adolescents and young adults with different chronic conditions (age = 12–31 years)	NR	The TRxANSITION Index	Administered by trained providers during routine visits or follow-up	Studies have shown that many AYAs rely on their caregivers for disease management, regardless of care setting, possibly explaining why they do not master these skills until an older age.
Culen et al. (55) Austria and Germany	To cross-culturally adapt and to pilot-test a German version of the Transition Readiness Assessment Questionnaire (TRAQ 5.0) and to provide a tool that can be applied broadly during the HCT process of YSHCN.	Pilot study	172 patients, YSHCN, with different chronic conditions (age = 14–23 years)	NR	TRAQ-GV-15	Self- administered, during routine clinical care	The German version of the TRAQ has a direct benefit for YSHCN. The administration of the TRAQGV−15’s inevitably led to transition centered communication with health professionals, encouraged caregivers to enhance AYAs’ autonomy and sensitized YSHCN for transition specific issues.
Shackleford et al. (40) United States	To examine the relationship between the three innate needs of Self-Determination Theory (SDT), self-management of care and adherence to treatment, and the relationship with health-related quality of life (HRQOL) for adolescents with congenital heart disease (CHD).	Experimental, cross-sectional, correlational study	92 patients with congenital heart disease (age = 13–18 years)	Autism, DiGeorge Syndrome	UNC TRxANSITION Scale	Self-reported	Better family and social support have been associated with better treatment adherence in adolescents with chronic illnesses, including those with renal, liver, heart, and lung transplants. Also, better peer support has been associated with improved adherence in adolescents with diabetes and asthma
Tan et al. (51) Malaysia	To assess medication self-management among parents of children with epilepsy and its association with sociodemographic data, clinical characteristics, antiepileptic drug (AED) regimen complexity, and parent self-reported AED adherence.	A cross-sectional survey	166 parents of children with epilepsy (age ≤ 18 years)	NR	Pediatric Epilepsy Medication Self-Management Questionnaire (caregiver version)	Self-administered, in hospital	The degree of medication self-management among parents of children with epilepsy was satisfactory. A more complex regimen was associated with poorer medication self-management. Barriers to treatment, including disliking taste, parent forgetfulness, and swallowing difficulties, should be addressed to empower parents in achieving better medication self-management.
Hart et al. (63) United States	To develop and evaluate a disease-neutral, parental report of their own health knowledge regarding their youth’s condition and a parental proxy assessment of youth HCT readiness to be used in parents of youth with chronic health conditions and verified against the medical record by adapting the TRxANSITION Index.	NR	93 parents of children with different chronic conditions (age = 12–25 years)	NR	TRxANSITION Index–Parent Version	Administered via personal interview and verified against the medical record	The TRxANSITION Index–Parent Version shows promise as a means of assessing parent knowledge of a youth’s illness and may provide an accurate proxy assessment of a youth’s HCT readiness skills.
Ma et al. (57) China	To translate, culturally adapt and evaluate the reliability and validity of the Chinese version of the Self-Management and Transition to Adulthood with Rx = Treatment Questionnaire	Multicenter cross-sectional	471 children and young people/adolescents with different chronic conditions (age = 8–18 years)	NR	Star X—C	Self-reported	Four major factors were identified in the Chinese version of the questionnaire, and it had a good fit to the target population. Validity was analyzed through EFA using principal component analysis with varimax rotation and CFA.

*According to the DSM 5 classification; NR, Not Reported; SCIS, Self-Care Independence Scale; TRAQ, Transition Readiness Assessment Questionnaire; CSU, Central South University; HCT, Health Care Transition; AYA, Adolescent and young adult; SHCN, Special Health Care Needs; GFI, Goodness of fit index; CFI, Comparative Fit Index; RMSEA, Root Mean Square Error of Approximation; ICC, Intraclass Correlation; CYP, Children and Young People; YSHCN, Youth with Special Health Care Needs; USA, United States of America.

Nine studies (39.13%) developed or used self-care instruments only for one type of chronic condition: spina bifida (*n* = 1; 4.76%) (46), type 1 diabetes (*n* = 3; 14.28%) (47–49), epilepsy (*n* = 2; 9.52%) (50, 51), asthma (*n* = 1; 4.76%) (52), cystic fibrosis (*n* = 1; 4.76%) (38), and congenital heart disease (*n* = 1; 4.76%) (40). Ten studies focused on multiple chronic conditions such as kidney disease, systemic lupus erythematosus, inflammatory bowel disease, hypertension, renal transplant, and systemic lupus erythematosus (2, 29, 53–61). Four studies did not specify the chronic condition of their sample (57, 62–64).

A total of 13 studies (56.52%) considered neurodevelopmental and/or mental disorders (according to the DSM 5 classification) as an exclusion criterion; two studies (8.69%) included also patients with neurodevelopmental and/or mental disorders, eight studies (34.78%) did not specify whether these disorders were considered exclusion criteria.

Twenty-two of the studies included in this review reported the administration method of the instruments. The authors of four studies (18.2%) specified that the questions were asked by an assistant researcher. With regard to data collection, seven studies (69.56%) used paper-and-pencil instruments administered in hospital settings, nine studies (39.13%) used online or telephone or mail interviews, while seven studies (69.56%) did not specify this. Most of the selected papers included information about the validity and reliability of the instruments (*n* = 20; 86.9%), while in three papers this information was not provided because they were based on previous validation studies.

### Characteristics of the Self-Care Instruments

Overall, 11 self-care instruments focusing on pediatric patients with chronic conditions were described in the studies included in this review (Table 2). Seven instruments were adapted, translated or modified from previous instruments developed by other authors (2, 40, 47, 51, 53, 55, 61). Five (45.45%) of the 11 instruments were specifically used to assess self-care of pediatric patients during the transition process from pediatric to adult care (29, 55, 59–61, 65). Five (45.45%) instruments were published in English (38, 46, 52, 58, 59); one instrument was available in English and Spanish (9.09%) (56); one was published both in English and German (9.09%) (65); two were available in English and Chinese (18.18%) (29, 49); one instrument was both in English and Italian (9.09%) (62), and one is available in English and Malaysian (9.09%) (50). Five (45.45%) instruments were entirely in line with the purposes of this review (38, 49, 58, 62, 65). Indeed, every dimension of the instruments included the concept of self-care and thus were analyzed in every part. The remaining instruments (*n* = 6; 54.54%) were analyzed only for those dimensions that were relevant to the aim of this review.

**TABLE 2:** Characteristics of the self-care measurement tools retrieved from this review.

Name of the measurement tool, Original/Adapted	Author and Year	Description of the tool or subscale	N. Included items	Languages	Complete tool or dimension	Target population and Chronic condition	Patient age (year)	Administration method	Conceptual model
Family Asthma Management System Scale (FAMSS) Original	Klinnert et al. (52)	The FAMSS evaluates the effectiveness of the family asthma management system.	11 subscales	English	4/11 dimensions: Adherence with asthma medications, Adherence with environmental recommendations, Assessment of child’s symptoms, Appropriateness of action plan and emotional response to asthma symptoms	Parents of children with asthma	6–9	Administered by research assistants	Family asthma management system
Self-Care Independence Scale (SCIS) Original	Patton et al. (38)	The SCIS evaluates children’s level of independence in their cystic fibrosis treatment.	44	English	Complete scale	Parents of children with cystic fibrosis	4–17	Self-reported	NR
Self-Management of Type 1 Diabetes in Adolescence (the SMOD-A) Original	Schilling et al. (49)^†^	The SMOD-A evaluates self-management in youths with type 1 diabetes.	33	English/Chinese^‡^	Complete scale	Patients with type 1 diabetes	13–21	Self-reported	NR
Pediatric Epilepsy Medication Self-Management Questionnaire (PEMSQ) Original	Modi et al. (50)	The PEMSQ evaluates medication self-management in children with epilepsy.	15 of 27	English/Malay^§^	2/4 dimensions: Adherence to medications and clinical appointments, Barriers to treatment	Parents of children with epilepsy	2-14	Self-reported	NR
Adherence Schedule in Transplantation-Proxy Child (ASiT-PC) Original	Giardini et al. (62)	The ASiT-PC evaluates the cognitive relational antecedents of adherence to treatment and the self-efficacy in disease management in patients before and after transplantation.	11	Italian^¶^	Complete scale	Parents of children, solid-organ pre and post-transplant recipients	NR	Self-reported	NR
Transition Readiness Assessment Questionnaire (TRAQ) Original	Sawicki et al. (65)mm	The TRAQ evaluates readiness for healthcare transition among youth with special healthcare needs.	20	English/German*	Complete scale	Patients with special health care needs and their parents	16–26	Self-reported	Transtheoretical model
(UNC) TR(x)ANSITION Scale Original	Ferris et al. (56)^#^	The (UNC) TR(x)ANSITION Scale evaluates self-management and health care transition skills in adolescents and young adults with chronic conditions.	13 of 33	English/Spanish^◆^	3/10 dimensions: Adherence, Nutrition, Self-management skills	Patients with chronic conditions	12–22	Administered by the transition coordinator through an interview	A report of academy of science and self-determination theory
Successful Transition to Adulthood with Therapeutics = Rx (STARx) Questionnaire Original	Ferris et al. (29)°	The STARx questionnaire evaluates health transition readiness in young people with chronic diseases.	9 of 18	English/Chinese^■^	Section 1 of 3	Patients with chronic conditions	8–25	Administered individually online	NR
ON Taking Responsibility for Adolescent/Adult Care (ON TRAC) Adapted from Williams et al. (92)	Moynihan et al. (58)	The revised ON TRAC evaluates youths’ capabilities in performing life skills required to actively participate in their health care and function independently as adults.	25	English	Complete scale	Patients with chronic conditions	12–19	NR	Holistic model
The Parent STARx Questionnaire (STARx-P) Adapted from Ferris et al. (29)	Nazareth et al. (59)	The STARx-P Questionnaire evaluates parents’ perspective on their child’s health care transition readiness.	5 of 18	English	1/3 dimension: self-management	Parents of children with chronic conditions	6–17	Self-reported, online	NR
Adolescent Self-Management and Independence Scale II (AMIS II) Original	Sawin et al. (46)	The AMIS II evaluates the adolescent’s increasing responsibility for and implementation of self-management behaviors.	5 of 17	English	2/8 dimensions: Self-management medications, Preventing complications	Patients with spina bifida and their parents	12–25	Structured interview rated by healthcare providers	NR

^†^The same scale is used by Karahroudy et al. (48) (Author of included study); ‡The Chinese version is C-SMODA by Guo et al. (47) (included study); §The Malay version is by Tan et al. (51) (included studies); ^¶^Translated in English; The same scale is also used by Sawicki et al. (60) (included study); *The German version is TRAQ G.V 15 by Culen et al. (55); ^#^The same scale is also used by Shackleford et al. (40) and Zhong et al. (61) (included studies); °The same scale is used by Cohen et al. (54) ◆ *The Spanish version is C TRxANSITION Scale™ Version 3 by Cantú-Quintanilla et al. (53) (included study);* ◼ *The Chinese version is by Sheng et al. (2) and Ma et al. (57) (included studies).*

The self-care instruments were developed for patients (*n* = 4; 36.36%) (49, 56, 58, 65), for parents (*n* = 5; 45.45%) (38, 50, 52, 59, 62), or both (*n* = 2; 18.18%) (46, 65). The target population of the instruments were adolescents/young adults (*n* = 4; 36.36%) (29, 46, 49, 56, 58, 65), children/adolescents (*n* = 4; 36.36%) (29, 50, 58, 59), children (*n* = 1; 9.09%) (52). One instrument (9.09%) (62) did not describe the target population. Five instruments (45.45%) were developed for specific conditions. In particular, these instruments were: the Self-Care Independence Scale (SCIS) for cystic fibrosis (38), FAMSS (38, 46, 49, 50, 52) the Pediatric Epilepsy Medication Self-Management Questionnaire (PEMSQ) (50), Self-Management of Type 1 Diabetes in Adolescence (SMOD-A) (49), the Adolescent Self-Management and Independence Scale II (AMIS II) (46). The remainder (*n* = 6.54; 54%) were developed for non-specific chronic conditions (29, 56, 58, 59, 62, 65). Five of the 11 instruments were self-administered (45.45%) (38, 49, 50, 62, 65), three instruments were administered by others, such as the research assistant (27.27%) (29, 46, 52, 56, 59) and one (9.09%) did not specify this (58).

Four of the 11 instruments (36.36%) described the conceptual models of reference, the other seven instruments (63.63%) did not refer to any conceptual model. In particular, the UNC TRxANSITION scale used the self-determination theory as reference model (56), the TRAQ scale used the Transtheoretical model (65), the Family Asthma Management System Scale (FAMMS) was developed according to the Family asthma management system model (52), and the ON Taking Responsibility for Adolescent/Adult Care (ON TRAC) used the Holistic model (58). *Psychometric characteristics of the tools (Validity and Reliability).*

The 13 studies reported in Table 3 explored the psychometric characteristics—validity and reliability—of the 11 measurement tools included in this review. Content validity was tested for five instruments (46, 49, 56, 57, 65), following the COSMIN taxonomy (45). Construct validity was verified through Exploratory Factor Analysis (EFA) for six instruments (29, 49, 50, 57–59, 65), and through Confirmatory Factor Analysis (CFA) for two instruments (46, 57). Only for the Star-x instrument the construct validity was verified both through EFA and CFA (57). With regard to criterion validity, concurrent validity was used to analyze four instruments (38, 46, 54, 58), and among these instruments, predictive validity was verified only for StarX and TRAQ (54, 65). The Known-groups validity was tested for the TRAQ instrument (65). Furthermore, the correlation/regression between self-care and child age was explored in seven instruments (29, 38, 50, 54, 56–59, 65).

**TABLE 3:** Psychometric characteristics of the self-care tools.

			VALIDITY						RELIABILITY		
References	Measurement tool	Face and Content validity	EFA	CFA	Known-groups validity (*p* < 0.05)	Correlation/Regression Self-care with child’s age (*p* < 0.05)	Concurrent validity	Predictive validity	Internal consistency	Test-retest	Inter-rater
Klinnert et al. (52)	Family Asthma Management System Scale (FAMSS)	NR	NR	NR	NR	NR	NR	NR	Cronbach’s alpha for FAMSS Summary Score was 0.91.	NR	Inter-rater reliability was assessed based on 15 (50%) interviews. Intraclass correlation for was high (*r* = 0.97).
Patton et al. (38)	Self-care Independence Scale (SCIS)	NR	NR	NR	NR	Scores correlated positively with child’s age (*r* = 0.67, *P* < 0.001).	Scores were related to children’s treatment knowledge and level of dependence in non-illness activities.	NR	Cronbach’s coefficient alpha: 0.93.	35 parents completed the SCIS twice over a 4-week period (*r* = 0.81, *P* < 0.001)	Interrater reliability, was good for both the total knowledge score (*r* = 0.93, *P* < 0.001) and individual treatment domain levels (*r* = 0.73–0.96, *P* < 0.01).
Schilling et al. (49)	Self-Management of Type 1 Diabetes in Adolescence (SMOD-A)	Assessed by multiple groups of experts.	EFA revealed five subscales	NR	NR	NR	NR	NR	Cronbach’s alphas for the five subscales ranged from 0.71 to 0.85.	Over 3 months it was 0.67 for the total scale and ranged from 0.34 to 0.47 for the 5 subscales	NR
Modi et al. (50)	Pediatric Epilepsy Medication Self-Management Questionnaire	NR	Principal axis factoring	NR	NR	No significant correlations were found with child’s age and Pediatric Epilepsy Self-Management scales.	NR	NR	Cronbach’s alphas ranged from 0.63 to 0.93.	NR	NR
Giardini et al. (62)	Adherence Schedule in Transplantation-Proxy Child (ASiT-PC)	NR	NR	NR	NR	NR	NR	NR	NR	NR	NR
Sawicki et al. (65)	Transition Readiness Assessment Questionnaire—TRAQ	Content/face validity by youths and experts	Principal component exploratory factor analysis.	NR	TRAQ scores differed based on the respondent’s primary diagnosis, age, and sex.	Older age was associated with higher scores (*p* < 0.001)	NR	Multivariate linear regression	Cronbach’s alpha for the entire questionnaire was: 0.93.	NR	NR
Ferris et al. (56)	UNC TRxANSITION Scale	Determined with national experts, clinicians, and patients.	NR	NR	NR	The total score increased with the advancing of age at univariate linear regression (β = 1.08, *p* < 0.0001).	NR	NR	Each of the items significantly correlated in a moderate to strong fashion (*r* > 0.42) with their respective domains (item-total correlations).	NR	Two independent members of the research team assessed the extent of agreement of the responses to the items (κ = 0.71, 95% CI: 0.64, 0.77).
Ferris et al. (29), Cohen et al. (54)	STARx	NR	EFA identified six factors that accounted for about 65% of the variance: Medication management, Provider communication, Engagement during appointments, Disease knowledge, Adult health responsibilities, and Resource utilization.	NR	NR	Participant age and STARX total score were positively correlated (*r* = 0.396, *p* < 0.001).	The STARx and its subscales positively correlated with the scores of the UNC TRxANSITION Scale™ and the TRAQ.	The STARx was correlated positively with the literacy, self-efficacy, and adherence measures.	Alpha coefficient for the overall scale: 0.80.	26 participants had two different test observations with 365 days: strong correlation between the first and second total score (β = 0.704, *p* < 0.001)	NR
Ma et al. (57)	STARx, Chinese version	The overall S-CVI/Ave of the expert content validity scores was 0.96	Four major factors were identified at EFA using principal component analysis with varimax rotation.	The CFA model indicated that there was a good fit.	NR	The *t*-test revealed that the scores of three factors and the overall scale were lower in the 8–11 than the 12–18 years age group.	NR	NR	Cronbach’s α = 0.812.	NR	NR
Nazareth et al. (59)	Parent STARx	NR	The factors of the STARx-P were confirmed using the same PCA method that was used to determine the underlying structure of the STARx.	NR	NR	The STARx-P self-management subscale was correlated positively with child chronological age.	NR	NR	Cronbach’s alpha coefficient was moderate to good (α = 0.545–0.759).	NR	NR
Moynihan et al. (58)	Am I ON TRAC	NR	EFA using PCA identified a 14-item unidimensional scale.	NR	NR	Knowledge and behavior sub-scale scores increased with age.	The Psychosocial Maturity Inventory had significant moderate correlations with the ON TRAC scores.	NR	For Cronbach’s alpha coefficient was 0.84.	NR	NR
Sawin et al. (46)	AMIS II	Content validity was conducted using health care professionals, parents, and AYA.	EFA, using principal axis analysis with an oblique rotation, conducted on parent data, supported two related self-management factors.	CFA of AYA data confirmed the two factors and an overall scale with good fit statistics.	NR	AYA age had low but significant correlations with Condition Self-Management (*r* = 0.24 and 0.30 for parent and AYA report) but higher relationships to Independent Living Self-Management (*r* = 0.047–0.54 for parent and AYA report).	Higher self-management behaviors were correlated with less severe SB (sacral lesions), better self-care, higher decision-making participation and maturity, participation in higher number of chores, and higher adolescent responsibility for overall condition management.	NR	Cronbach’s alpha for the scale and subscales was 0.78–0.82. Cronbach’s alphas for parent and adolescent was 0.87–0.89 for the total scale.	43 parents-AYA pairs, at a 2–3 week interval: ICC for parent total scale was 0.82, and for the AYA total scale was 0.84.	Inter-rater reliability with the first author was established (*r* = 0.88). Periodical confirmation of inter-rater reliability (*r* = 0.85).

*AYA, Adolescent and young adults; RMSEA, Root Mean Square Error of Approximation; GFI, Goodness of fit index; CFI, Comparative Fit Index; EFA, Exploratory factor analysis; PCA, Principal Component Analysis.*

Regarding reliability, internal consistency was verified in 11 instruments (29, 38, 46, 49, 50, 52, 56–59, 65). Test-retest reliability was tested in four instruments (29, 38, 46, 49), and inter-rater reliability was also verified for the SCIS e the AMIS II scale (38, 46). With regard to the FAMSS and the UNC TRxANSITION Scale, inter-rater reliability was verified in addition to internal consistency (52, 56). Lastly, responsiveness and non-differential validity were not reported for any instruments.

### Self-Care Aspects of Each Instrument

In this review, the self-care aspects/areas reported in the 11 instruments were described according to Riegel’s self-care theory focusing on the aspects of each of the three self-care domains (self-care maintenance, self-care monitoring, self-care management) by Riegel (Table 4). As regards to self-care maintenance, all the 11 instruments included the aspect/area of medication adherence and only the (UNC) TR(x)ANSITION Scale and The Parent STARx Questionnaire (STARx-P) included the aspect of treatment adherence (56, 59). Feeding was explored in three instruments: the UNC Tr(x)ansition scale, Transition Readiness Assessment Questionnaire (TRAQ) and Adherence Schedule in Transplantation-Proxy Child (ASiT-PC) (56, 62, 65). The aspect of lifestyle was examined only in the TRAQ (65), whereas the prevention aspect was included in two instruments: FAMSS and ON TRAC (52, 58). The knowledge of health-care services was explored in two instruments, the Parent STARx Questionnaire (STARx-P) and TRAQ (59, 65).

**TABLE 4:** Self-care dimensions and aspects of each measurement tool.

		Self-care maintenance						Self-care monitoring		Self-care management
Name of the measurement tool	References	Medication adherence	Treatment	Nutrition	Life style	Prevention	Knowledge of health-care services	Clinical parameters	Symptoms/Signs	Consulting
FAMSS	Klinnert et al. (52)	X				X				
SCIS	Patton et al. (38)	X								
SMODA	Schilling et al. (49)	X					X	X		X
PEMSQ	Modi et al. (50)	X								
ASiT-PC	Giardini et al. (62)	X		X				X		
TRAQ	Sawicki et al. (65)	X		X	X		X			
(UNC) TR(x)ANSITION Scale	Ferris et al. (56)	X	X	X						X
Rx STARx	Ferris et al. (29)	X								X
ON TRAC	Moynihan et al. (58)	X				X				X
AMIS II	Sawin et al. (46)	X						X	X	
STARx-P	Nazareth et al. (59)	X	X				X			X

*TRAQ, Transition Readiness Assessment Questionnaire; SCIS, Self-Care Independence Scale; ASIT-PC, Adherence Schedule in Transplantation-Proxy Child; FAMSS, Family Asthma Management System Scale; PEMSQ, Pediatric Epilepsy Medication Self-Management Questionnaire; Rx STARx Questionnaire, Successful Transition to Adulthood with Therapeutics; STARx-P, The Parent STARx Questionnaire; ON TRAC, ON Taking Responsibility for Adolescent/Adult Care; SMODA, Self-Management of Type 1 Diabetes in Adolescence; C SMOD-A, Chinese Version of Self-Report Measure of Self-Management of Type 1 Diabetes for Adolescents; AMIS II, Adolescent Self-Management and Independence Scale II.*

With regard to self-care monitoring, the area of vital signs monitoring was explored only in the Adherence Schedule in Transplantation-Proxy Child (ASiT-PC) and in the Adolescent Self-Management and Independence Scale II (AMIS II) (46, 62). The signs and symptoms aspect was treated in the Self-Management and Independence Scale II (AMIS II) (46).

For the self-care management domain, only one aspect (i.e., consulting) was described in four instruments: Successful Transition to Adulthood with Therapeutics = Rx (STARx) Questionnaire, (UNC) TR(x)ANSITION Scale, ON TRAC and The Parent STARx Questionnaire (STARx-P) (29, 56, 58, 59).

## Discussion

In this literature review, 11 self-care instruments addressing CYAs with chronic diseases were identified. These instruments differ for pathologic contexts and age range. The instruments providing more valid psychometric measurements were AMIS II and STARx. These are also the instruments published more recently (46, 57).

Although all the identified instruments included at least one of Riegel’s self-care domains, only one instrument, the SMODA, investigated all of the three self-care domains: self-care maintenance, self-care monitoring, self-care management (49). Every instrument included in this review explored self-care maintenance, focusing particularly on medication and treatment adherence. The importance attributed to autonomy in medication administration might be associated to the advantage of reducing use of healthcare services (66). However, few instruments focused on monitoring vital signs and symptom management. Concerning self-care monitoring, only one instrument investigated signs and symptoms monitoring, an important aspect to detect important health status changes. As regards the self-care management area, the instruments explored mostly the consulting aspect, leaving very little space for the management of complications or acute exacerbations through spontaneous self-care strategies. Overall, the medical management of a chronic condition is not new, probably along with paternalistic and directive guidance in the relationship between healthcare providers and families (67), often associated with the passive decision-making of families allowing the provider to choose the course of action (68, 69). This aspect might reflect the persisting belief in the monopoly on health of the healthcare providers (70), and a great trust assigned to healthcare professionals of reference, such as the nurse case managers (71).

Studies showed that also self-care monitoring and self-care management is important. For example, Riegel et al. (27) describe how symptom monitoring affects self-care behaviors, underling the importance of symptom detection, interpretation and response as fundamental elements of the self-care process (27). Sawin et al. (46) found that when CYAs monitored their signs and symptoms they achieved independence much earlier than others (46). Also Nazareth et al. (59) found that when CYAs responded promptly to signs and symptoms of exacerbation they became more knowledgeable about their disease management (59).

Specific instruments were developed for the most common chronic diseases. In particular, the SMOD-A scale was developed for diabetes, SCIS for cystic fibrosis, FAMSS for asthma, PEMSQ for epilepsy, and AMIS II for spina bifida (38, 46, 49, 50, 52). The decision to have an instrument for a specific disease might be due to the large prevalence of these diseases, mostly diabetes and asthma, in the CYA population (72, 73). However, even though having instruments for assessing self-care in CYAs with specific chronic diseases is fundamental, there are also many other chronic and sometimes rare conditions to take into consideration. Therefore, the development of a non-specific instrument for the CYA population with different chronic conditions, considering the main age stages, might represent a useful innovation.

Furthermore, in this review, two instruments were found to be more generic [i.e., (UNC) TR(x)ANSITION Scale and TRAQ)] designed for chronic diseases in general or young adults with special healthcare needs (56, 65). However, these two instruments were focused on the skills required during the healthcare transition from pediatric to adult care services. Transition readiness reflects all the indicators (e.g., disease-specific knowledge, scheduling appointments) that young adults can begin, continue, and finish the transition process, including those skills influencing self-care (74–77). Therefore, self-care might be considered an integral part of transition readiness in the context of a challenging transfer to the adult health care system. To our knowledge, no instrument is currently available to assess self-care behaviors among CYAs of all ages aimed at exploring the shift of agency from family to autonomous self-care, regardless of the patient care context.

Moreover, also complex chronic diseases need to be considered. According to Cohen et al. (15), these conditions in childhood are characterized by four domains: (a) family-identified healthcare service needs, (b) one or more chronic clinical conditions, either diagnosed or unknown, (c) severe functional limitations, and (d) highly projected utilization of health resources. CYAs with complex chronic conditions need standardized approaches, tools and more effective competence to manage the complexity of these diseases (78). Therefore, it would be useful to develop a specific self-care instrument for CYAs with complex chronic conditions.

Furthermore, the present review analyzed also the methods used to administer the instruments. Five instruments were self-reported since the respondents were in school-age or adolescents (38, 49, 50, 62, 65). Four instruments asked also the parents to fill in the questionnaire. This aspect might show how the family maintain a central and vital role in supporting both the children—during the pre and scholar age—and adolescents/young adults (79, 80).

Another aspect analyzed in the current review was the conceptual model underpinning each instrument. The conceptual models were specified in four instruments, such as the self-determination theory, the transtheoretical model, the holistic model, and family management of specific diseases such as asthma. Having instruments based on a theoretical framework, as the models reported above, is fundamental to obtain sounder and valid instruments (81). Recently, a self-care model has been developed for complex chronic conditions (28). This model includes the affecting factors, self-care behaviors and outcomes, highlighting that the more the patients are engaged in self-care behaviors, the more the results are positive.

### Implications for Practice

The findings of this review may help researchers identify instruments to assess the level of self-care in CYAs living with chronic conditions for research purposes, as outcome measures for interventional studies, or as a basis for further validation studies. Moreover, we recommend the use of self-care instruments in clinical practice. Although clinicians recognize the importance of promoting self-care in CYAs with chronic conditions, they need standardized approaches and psychometrically sound tools (78). Measuring a patient’s level of self-care using an assessment instrument represents, for the clinician, the first step to identifying educational gaps and factors hindering the engagement process (82). Healthcare providers play an important role in fostering autonomy using educational strategies that take into consideration developmental stages and family support (83–85). Educational interventions have resulted in improvements in health outcomes, knowledge related to the chronic condition, quality of life, attendance at school, participation in social activities, and a decrease in health service interactions (86–88). In a second step, the patient could assume greater responsibility for managing their health alone or with the help of parents and healthcare professionals (89).

### Limitations

The findings of this review should be considered in light of some limitations. Firstly, the current review explored the instruments that concerned self-care in CYAs in every context. However, these contexts were found to be very broad and the concept of self-care may overlap with other concepts such as healthcare transition from pediatric to adult clinical setting (56). Therefore, the instruments found could not be considered totally comparable.

Secondly, the search strategy of this review did not include gray literature. Therefore, unquantifiable instruments might have been missed. Thirdly, studies measuring self-care in CYAs with neurocognitive impairment and in those living with cancer were not included in this review. Although the authors of this review believe that these patients deserve specific considerations, important features of the self-care process may have been missed.

Another limitation was the voluntary exclusion of the studies that assessed self-efficacy. Indeed, the aim of this review was to identify the instruments that evaluate self-care behaviors (maintenance, monitoring, management) rather than assessing confidence in self-care (90). However, future quantitative studies could investigate the confidence aspect since the process of self−care implies that self−care confidence (in patients and caregivers) influences the entire process of self−care across the three dimensions of self−care maintenance, monitoring, and management (91).

## Conclusion

This review analyzed 23 studies that described 11 self-care instruments for CYAs. Only one instrument assessed each aspect of self-care (maintenance, monitoring, and management) according to our definition. In particular, most of the instruments were focused on treatment adherence within self-care maintenance and ignored the aspects of prevention, feeding, and lifestyle. Less attention was given to vital signs and symptoms monitoring, and to responses to exacerbations of chronic conditions. Therefore, it would be useful to investigate how health professionals are focused on these self-care dimensions while providing education to patients and their families. Furthermore, future research may develop a comprehensive instrument measuring all the dimensions of self-care across all chronic conditions, also including those with medical complexity. Future instruments might be based on “The comprehensive model of self-care in CYA with chronic conditions” (28). This model could guide to a global evaluation of self-care in relation to developmental age, also considering the parent’s contribution and shift of agency.

## Data Availability Statement

The original contributions presented in the study are included in the article/Supplementary Material, further inquiries can be directed to the corresponding author/s.

## Author Contributions

GS, VB, and VK made substantial contributions to the conception and design of the systematic review, conducted the literature search, data extraction, and drafted the manuscript. RM gave a substantial contribution to the translation of the text, moreover, critically reviewed and revised the manuscript. AL and GM contributed to evaluated the psychometric proprieties of the tools retrieved, updating the reference list, critically reviewed, and revised the manuscript. OG, MS, EV, and ET helped in results analysis, drafting and critically revising the manuscript. ID’O conceived and supervised all the phases of the systematic review, drafted and critically revised the manuscript for important intellectual content. All authors approved the final version of the manuscript as submitted and agreed to be accountable for all the aspects of this study.

## Conflict of Interest

The authors declare that the research was conducted in the absence of any commercial or financial relationships that could be construed as a potential conflict of interest.

## Publisher’s Note

All claims expressed in this article are solely those of the authors and do not necessarily represent those of their affiliated organizations, or those of the publisher, the editors and the reviewers. Any product that may be evaluated in this article, or claim that may be made by its manufacturer, is not guaranteed or endorsed by the publisher.
