# Supplementary material for: Instruments Measuring Self-Care in Children and Young Adults With Chronic Conditions: A Systematic Review
Source: Front Pediatr. 2022 Mar 28;10:832453. doi: 10.3389/fped.2022.832453 (PMC8995847; doi:10.3389/fped.2022.832453)
Supplement: Supplementary file 1 [file Data_Sheet_1.PDF]

Supplementary file - Search strategy

I. PubMed 650 Results (04/07/2021)

**Filters**	**Search statement**
All fields	#6 "self care" OR "self-care" OR "selfcare" OR "self management" OR "self-management" OR complian* OR adheren*
All fields	#5 Scale* OR "Rating scale*" OR "Behavior Rating Scale" OR “Behaviour Rating Scale” OR "Evaluation tool" OR "Evaluation* tool*" Instrument* OR tool OR Questionnaires OR Questionnaire*
all fields	#4: #1 AND #2 NOT #3
all fields	#3: "Diagnostic and Statistical Manual of Mental Disorders" OR "Neoplasms" OR "Hematologic Neoplasms" OR "Terminal Care"
all fields	#2: "Chronic Disease" OR "Chronic Disease*" OR "Chronic* Ill* "OR "Disease Chronic" OR "Chronic Illness" OR "Chronic* Illness*" OR "Illness Chronic" OR "Chronically Ill" OR "Diseases Chronic" OR "Chronic Illnesses" OR "Complex conditions" OR "multiple chronic conditions" OR multimorbidity OR "Long-term Care" OR "Long term conditions" OR "Long term condition*" OR "Long-term illness" OR "Long-term illnesses" OR "Long-term condition"
title/abstract	#1: Pediatr* OR Paediatr* OR Newborn OR Newborn* OR Infant OR Infant* OR Child OR Child* OR Preschool OR adolescent OR adolescen* OR teenager* OR teen-ager* OR “young adult” OR “young adult*”
/	#4 AND #5 AND #6

II. Scopus 297 Results (04/07/2021)

**Filters**	**Search statement**
title/abstract/keywords	"self care" OR "self-care" OR ‘’selfcare’’ OR "self management" OR "self-management" OR complian* OR adheren*
All fields	#5 Scale* OR "Rating scale*" OR "Behavior Rating Scale" OR “Behaviour Rating Scale” OR "Evaluation tool" OR "Evaluation* tool*" Instrument* OR tool OR Questionnaires OR Questionnaire*
title/abstract/keywords	#4: #1 AND #2 NOT #3
title/abstract/keywords	#3: "Diagnostic and Statistical Manual of Mental Disorders" OR "Neoplasms" OR "Hematologic Neoplasms" OR "Terminal Care"
title/abstract/keywords	#2: "Chronic Disease" OR "Chronic Disease*" OR "Chronic* Ill* "OR "Disease Chronic" OR "Chronic Illness" OR "Chronic* Illness*" OR "Illness Chronic" OR "Chronically Ill" OR "Diseases Chronic" OR "Chronic Illnesses" OR "Complex conditions" OR "multiple chronic conditions" OR multimorbidity OR "Long-term Care" OR "Long term conditions" OR "Long term condition*"OR "Long-term illness" OR "Long-term illnesses" OR "Long-term condition"
Title/abstract/keywords	#1: Pediatr* OR Paediatr* OR Newborn OR Newborn* OR Infant OR Infant* OR Child OR Child* OR Preschool OR adolescent OR adolescen* OR teenager* OR teen-ager* OR “young adult” OR “young adult*”
/	#4 AND #5 AND #6

I. CINHAL: 724 Results (04/07/2021)

Filters	Search statement
All fields	#6 "self care" OR "self-care" OR "selfcare" OR "self management" OR "self-management" OR complian* OR adheren*
All fields	#5 Scale* OR "Rating scale*" OR "Behavior Rating Scale" OR “Behaviour Rating Scale” OR "Evaluation tool" OR "Evaluation* tool*" Instrument* OR tool OR Questionnaires OR Questionnaire*
all fields	#4: #1 AND #2 NOT #3
all fields	#3: "Diagnostic and Statistical Manual of Mental Disorders" OR "Neoplasms" OR "Hematologic Neoplasms" OR "Terminal Care"
all fields	#2: "Chronic Disease" OR "Chronic Disease* " OR "Chronic* Ill* "OR "Disease Chronic" OR "Chronic Illness" OR "Chronic* Illness* " OR "Illness Chronic" OR "Chronically Ill" OR "Diseases Chronic" OR "Chronic Illnesses" OR "Complex conditions" OR "multiple chronic conditions" OR multimorbidity OR "Long-term Care" OR "Long term conditions" OR "Long term condition*" OR "Long-term illness" OR "Long-term illnesses" OR "Long-term condition"
All fields	#1: Pediatr* OR Paediatr* OR Newborn OR Newborn* OR Infant OR Infant* OR Child OR Child* OR Preschool OR adolescent OR adolescen* OR teenager* OR teen-ager* OR “young adult” OR “young adult*”
/	#4 AND #5 AND #6

I. EMBASE 897 Results (04/07/2021)

Filters	Search statement
All fields	#6 "self care" OR "self-care" OR "selfcare" OR "self management" OR "self-management" OR complian* OR adheren*
All fields	#5 Scale* OR "Rating scale*" OR "Behavior Rating Scale" OR “Behaviour Rating Scale” OR "Evaluation tool" OR "Evaluation* tool*" Instrument* OR tool OR Questionnaires OR Questionnaire*
all fields	#4: #1 AND #2 NOT #3
all fields	#3: "Diagnostic and Statistical Manual of Mental Disorders" OR "Neoplasms" OR "Hematologic Neoplasms" OR "Terminal Care"
all fields	#2: "Chronic Disease" OR "Chronic Disease*" OR "Chronic* Ill* " OR "Disease Chronic" OR "Chronic Illness" OR "Chronic* Illness*" OR "Illness Chronic" OR "Chronically Ill" OR "Diseases Chronic" OR "Chronic Illnesses" OR "Complex conditions" OR "multiple chronic conditions" OR multimorbidity OR "Long-term Care" OR "Long term conditions" OR "Long term condition*" OR "Critical illness" OR "Long-term illness" OR "Long-term illnesses" OR "Long-term condition"
title/abstract	#1: Pediatr* OR Paediatr* OR Newborn OR Newborn* OR Infant OR Infant* OR Child OR Child* OR Preschool OR adolescent OR adolescen* OR teenager* OR teen-ager* OR “young adult” OR “young adult*”
/	#4 AND #5 AND #6

I. PsycInfo 216 Results (04/07/2021)

Filters	Search statement
All fields	#6 "self care" OR "self-care" OR selfcare OR "self management" OR "self-management" OR complian* OR adheren*
All fields	#5 Scale* OR "Rating scale*" OR "Behavior Rating Scale" OR "Behaviour Rating Scale" OR "Evaluation tool" OR "Evaluation* tool*" Instrument* OR tool OR Questionnaires OR Questionnaire*
all fields	#4: #1 AND #2 NOT #3
all fields	#3: "Diagnostic and Statistical Manual of Mental Disorders" OR "Neoplasms" OR "Hematologic Neoplasms" OR "Terminal Care"
all fields	#2: "Chronic Disease" OR "Chronic Disease*" OR "Chronic* Ill* "OR "Disease Chronic" OR "Chronic Illness" OR "Chronic* Illness*" OR "Illness Chronic" OR "Chronically Ill" OR "Diseases Chronic" OR "Chronic Illnesses" OR "Complex conditions" OR "multiple chronic conditions" OR multimorbidity OR "Long-term Care" OR "Long term conditions" OR "Long term condition*" OR "Long-term illness" OR "Long-term illnesses" OR "Long-term condition"
Title	#1: Pediatr* OR Paediatr* OR Newborn OR Newborn* OR Infant OR Infant* OR Child OR Child* OR Preschool OR adolescent OR adolescen* OR teenager* OR teen-ager* OR “young adult” OR “young adult*”
/	#4 AND #5 AND #6

I. Cochrane **542 Results (04/07/2021)**

Filters	Search statement
All fields	#6 "self care" OR "self-care" OR selfcare OR "self management" OR "self-management" OR complian* OR adheren*
All fields	#5 Scale* OR "Rating scale*" OR "Behavior Rating Scale" OR “Behaviour Rating Scale” OR "Evaluation tool" OR "Evaluation* tool*" Instrument* OR tool OR Questionnaires OR Questionnaire*
all fields	#4: #1 AND #2 NOT #3
all fields	#3: "Diagnostic and Statistical Manual of Mental Disorders" OR "Neoplasms" OR "Hematologic Neoplasms" OR "Terminal Care"
all fields	#2: "Chronic Disease" OR "Chronic Disease*" OR "Chronic* Ill* " OR "Disease Chronic" OR "Chronic Illness" OR "Chronic* Illness*" OR "Illness Chronic" OR "Chronically Ill" OR "Diseases Chronic" OR "Chronic Illnesses" OR "Complex conditions" OR "multiple chronic conditions" OR multimorbidity OR "Long-term Care" OR "Long term conditions" OR "Long term condition*" OR "Long-term illness" OR "Long-term illnesses" OR "Long-term condition"
All fields	#1: Pediatr* OR Paediatr* OR Newborn OR Newborn* OR Infant OR Infant* OR Child OR Child* OR Preschool OR adolescent OR adolescen* OR teenager* OR teen-ager* OR “young adult” OR “young adult*”
In Cochrane Reviews (Word variations have been searched)	#4 AND #5 AND #6
